# Modeling the association between illiteracy and poverty in Egypt: a comparative analysis of linear regression and ARDL approaches

**DOI:** 10.1038/s41598-026-47365-1

**Published:** 2026-04-18

**Authors:** Maha Mohamed Alsebai Mohamed, Alsebai Mohamed

**Affiliations:** Department of Economic, Faculty of Commerce, Benha University, Benha, Egypt

**Keywords:** Poverty Dynamics, Illiteracy Rate, Autoregressive Distributed Lag (ARDL), Egypt, Time-Series Analysis, Long-Run Relationship, Health care, Mathematics and computing

## Abstract

This study examines the dynamic relationship between illiteracy and poverty in Egypt over the period 1990–2023 using annual time-series data. The analysis applies both a simple linear regression model and the Autoregressive Distributed Lag (ARDL) framework to distinguish between short-run dynamics and potential long-run relationships. While the linear regression results indicate a statistically significant association between illiteracy and poverty, the ARDL (1,2) specification provides a more appropriate framework for capturing temporal adjustments and dynamic interactions. The ARDL bounds test yields inconclusive evidence regarding long-run cointegration, suggesting that the existence of a stable long-run equilibrium relationship cannot be confirmed with certainty. Consequently, long-run estimates are interpreted as indicative of potential sustained effects rather than definitive equilibrium outcomes. In contrast, the short-run results reveal statistically significant and cumulative effects of illiteracy on poverty. The error correction term is negative and statistically significant (ECM (− 1) = − 0.199), indicating that approximately 19.9% of short-term deviations from the long-run path are corrected each year. Diagnostic and stability tests confirm the robustness and validity of the estimated model. Overall, the findings highlight the importance of short-run dynamics and emphasize education as a critical policy instrument for poverty reduction and sustainable development in Egypt. By focusing on illiteracy as a key determinant, this study provides new empirical insights into the dynamic education–poverty nexus in a developing country context.

## Introduction

**Fig. 1:**
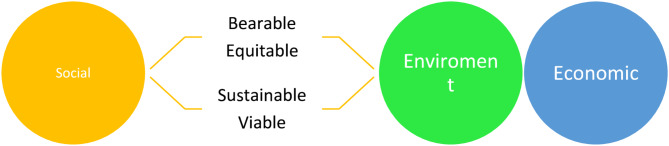
The Three dimensions of sustainable development and their intersections. Source: Adapted from: Barbier, E. (1987). *The Concept of Sustainable Economic Development*. Environmental Conservation, 14(2), 101–110.

Sustainable development integrates social, environmental, and economic dimensions to optimize resource use while meeting present needs without compromising the ability of future generations to meet their own^1,2^. Addressing environmental degradation alongside economic growth, equity, and social justice remains a central global challenge. As illustrated in Fig. 1, sustainability emerges from the interaction of three core pillars: economic growth, environmental conservation, and social development whose intersection represents balanced development consistent with the Sustainable Development Goals (SDGs)^3,4^.

Figure 1 presents the conceptual framework of sustainable development, where social equity, environmental protection, and economic viability interact. Their overlaps reflect key sustainability conditions: Bearable (social–environmental), Equitable (social–economic), and Viable (economic–environmental). Sustainability lies at the intersection of all three dimensions, representing the balanced coexistence of economic growth, social well-being, and environmental integrity.

**Fig. 2:**
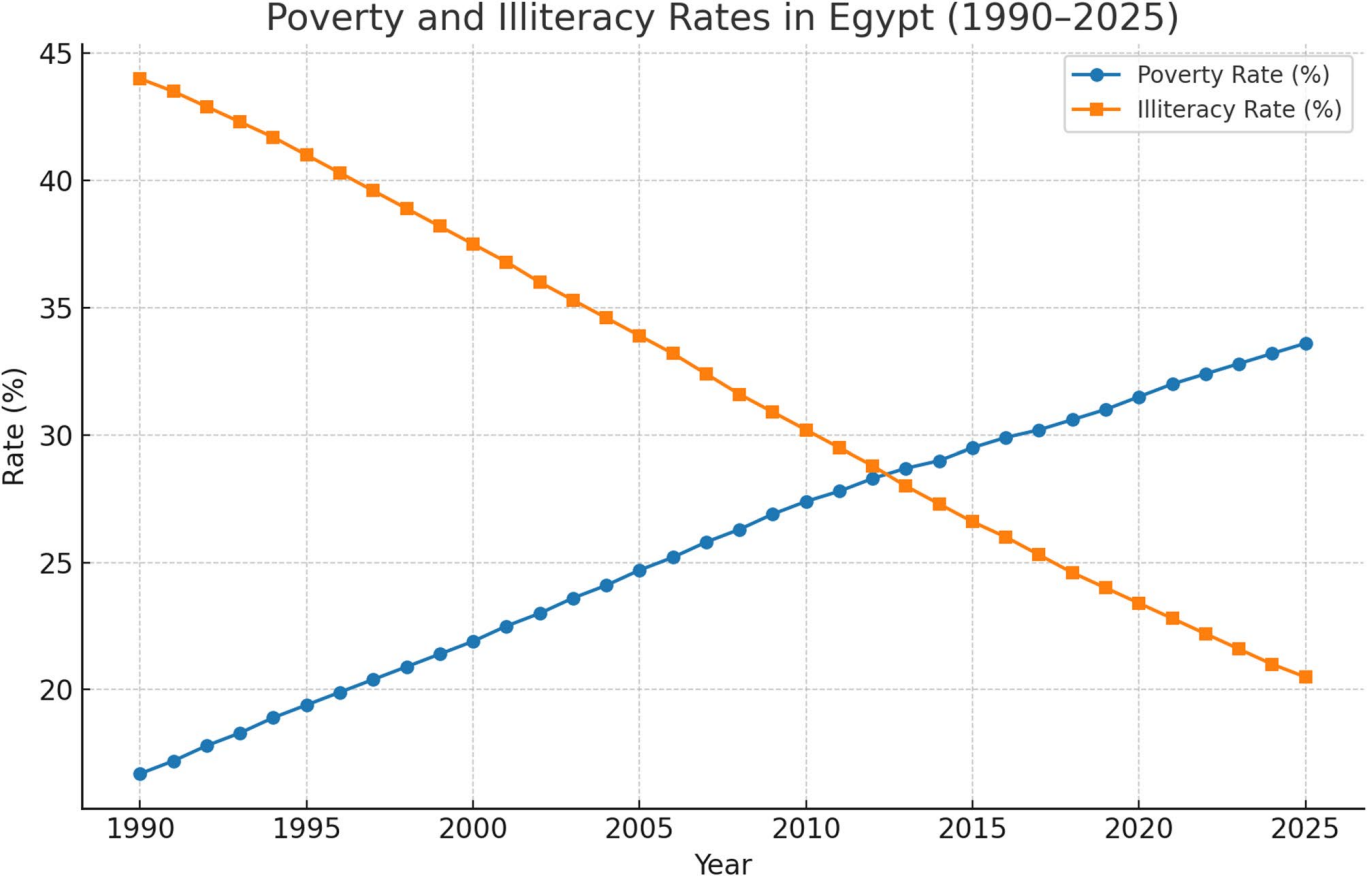
Descriptive Analysis of Poverty and Illiteracy in Egypt (1990–2025). Source: Prepared by the author based on statistical data on poverty in Egypt during the period from 1990 to 2025.

Poverty eradication constitutes a major challenge to sustainable development, requiring balanced production and consumption patterns without excessive reliance on natural resources. Poverty affects all countries, with substantial variation between advanced and developing economies^5,6^. In many African economies, illiteracy represents a persistent structural constraint, reinforcing poverty and limiting economic participation. In this regard, Tessema and Geda utilized dominance analysis to demonstrate that education and household wealth are the most influential drivers of socio-economic inequalities in developing contexts. Their findings support the prioritization of educational attainment as a primary intervention for social welfare^7^. Restricted access to basic education weakens human capital formation, while literacy initiatives contribute to improved livelihoods and sustainable development outcomes^8,9^. In Egypt, illiteracy is shaped by interconnected social, economic, cultural, and political factors and persists despite prolonged governmental and societal efforts^10,11^. In this context, Jin et al. (2025) emphasize that the success of these social efforts is often linked to optimal decentralization of spending, which is essential for achieving the Sustainable Development Goals and ensuring that public resources effectively reach the most vulnerable groups^12^. According to the Central Agency for Public Mobilization and Statistics, the national poverty rate reached 32% in 2017/2018, declining in 2019/2020 following the implementation of sustainable development initiatives Fig. 2. Over the period 2010–2020, Egypt experienced notable economic growth, with GDP increasing from USD 218.9 billion to USD 303 billion and per capita GDP rising from USD 2,646 to USD 3,548, while the Gini index remained consistently low^13–15^. Figure 2 illustrates the trajectories of poverty and illiteracy rates in Egypt from 1990 to 2025, highlighting their long-term trends and interrelationship.

Figure 2 illustrates the long-term trajectories of poverty and illiteracy rates in Egypt from 1990 to 2025. The data indicate a general decline in illiteracy rates, reflecting progress in educational outcomes, while poverty rates fluctuated in response to economic conditions but showed improvement in recent years. The narrowing gap between poverty and illiteracy suggests a potential association between educational progress and poverty reduction, without implying direct causality. These dynamics underscore the need for integrated social and economic policies. Development and poverty exhibit an inverse relationship, whereby higher levels of sustainable development are associated with reduced deprivation, although the magnitude and direction depend on labor market conditions, income distribution, and social policies^16,17^. Employment stability remains critical: in 2015/2016, 36% of the working poor held temporary jobs with a poverty rate of 27.8%, increasing to 38% temporary employment and 29.7% poverty in 2017/2018^18–20^. Despite global literacy initiatives, including the United Nations Literacy Decade (2003–2012), nearly one billion adults remain illiterate worldwide 600 million of whom are women concentrated in countries such as Bangladesh, Brazil, China, Egypt, India, Indonesia, Mexico, Nigeria, and Pakistan^21,22^.

Egypt’s Sustainable Development Strategy 2030, launched in 2016, outlines a comprehensive vision for economic, social, and environmental development, emphasizing improved quality of life, social justice, inclusion, and protection of vulnerable groups through health care, employment generation, and poverty alleviation programs^23,24^. Education and poverty reduction remain mutually reinforcing policy priorities, Limited literacy constrains human capital development and labor market opportunities, while poverty restricts access to education, reinforcing illiteracy^25–27^. Empirical evidence indicates that 35.6% of illiterate individuals were poor, compared to 9.4% of university graduates in the same year^28^.Illiteracy therefore represents a critical barrier to economic progress and comprehensive sustainable development^29–31^. However, addressing this obstacle requires not only educational reforms but also a balanced fiscal framework. As Jin et al. (2025) noted, sustainable development outcomes improve significantly when fiscal policies and spending structures are optimized to support social equity^12^.

Although the relationship between education and poverty is often portrayed as an inverse linear relationship, recent empirical and theoretical studies suggest that socioeconomic relations may exhibit nonlinear dynamics, threshold behavior, or asymmetric responses. In other words, a decrease in literacy rates may not lead to a proportional decrease in poverty rates at all stages of development. Rather, this effect may become more pronounced once literacy rates reach certain critical levels or in the presence of complementary economic conditions. Similar systemic perspectives are observed in interdisciplinary research, where complex systems exhibit feedback mechanisms and nonlinear responses among interacting variables (Liu et al., 2023)^32^. In socioeconomic contexts, systematic approaches such as structural equation modeling and systems-based frameworks have also been used to observe multidimensional relationships between social variables and performance outcomes^33^. These insights highlight the importance of considering potential nonlinearity and systemic interactions when studying the relationship between education and poverty.

Against this background, the present study examines the empirical association between illiteracy and poverty in Egypt over the period 1990–2023. The increased focus on illiteracy is further justified by the dominance of educational factors in determining life outcomes, as Tessema and Geda demonstrated, making it a crucial starting point for analyzing poverty dynamics^12^. This study employs both simple linear regression and the Autoregressive Distributed Lag (ARDL) approach. (ARDL) approach. Prior studies show that economic growth alone does not necessarily reduce poverty. Ravallion^34^ and Bourguignon^35^ emphasize the role of inequality and distributional effects, while Chenery et al.^36^ and Adams^37^ demonstrate that growth benefits are not uniformly shared. In the Egyptian context, Kheir El-Din and El-Laithy^38^ find that growth alone is insufficient for poverty reduction, underscoring the importance of income distribution and social protection. These findings motivate the present analysis and caution against simplistic interpretations of the poverty education relationship.

### Research problem

Poverty in Egypt is shaped by multiple socio-economic determinants, among which illiteracy plays a critical role. While existing studies have explored this relationship using static econometric techniques, such approaches often fail to capture dynamic adjustments and potential long-run associations, particularly in the presence of short-run fluctuations and structural changes. The focus on illiteracy is further supported by the dominance of educational factors in determining life outcomes, as identified by Tessema and Geda^12^. Accordingly, this study addresses the following research question:

Does the Autoregressive Distributed Lag (ARDL) model provide a more reliable characterization of the short-run dynamics and potential long-run association between poverty and illiteracy in Egypt compared to simple linear regression?

### Justification of the study

This research is closely aligned with the United Nations Sustainable Development Goals, particularly SDG 1 (No Poverty) and SDG 4 (Quality Education), By isolating the impact of illiteracy, the study recognizes it as a dominant driver a concept reinforced by Tessema and Gida (2023) and thus provides a focused benchmark necessary for targeted policymaking within the framework of Egypt’s Vision 2030^7^.which emphasize poverty reduction and educational improvement. Understanding the dynamic interaction between poverty and illiteracy is therefore essential for designing effective, evidence-based policies in Egypt. Most prior studies rely on static models that may yield biased or incomplete insights into this relationship. By applying the ARDL bounds testing approach, this study allows for the examination of short-run dynamics and the assessment of potential long-run associations, thereby addressing an important methodological gap in the existing literature and contributing to a more nuanced empirical understanding of poverty–education linkages.

### Research objectives

This study aims to:

1. Examine the trends of poverty and illiteracy rates in Egypt within the context of sustainable development, using descriptive analysis to provide an empirical overview.
2. Assess and compare the performance of simple linear regression and ARDL models in capturing the relationship between poverty and illiteracy.
3. Investigate the short-run dynamics and potential long-run associations between illiteracy and poverty rates in Egypt using the ARDL methodology.
4. Evaluate the robustness and stability of the ARDL estimates through standard diagnostic and stability tests to ensure reliable interpretation of the results.

## Research hypotheses and conceptual framework

### Research hypotheses

Based on the theoretical framework and economic references, literacy is considered a key factor in reducing poverty and improving economic opportunities. Therefore, this research focuses on the associational economic relationship between literacy rates and poverty levels. The hypotheses are formulated as follows:

#### H1

There is a statistically significant inverse relationship between illiteracy and poverty in Egypt; that is, a decrease in illiteracy rates is associated with a decrease in poverty levels.

#### H2

Illiteracy rates have a time-related impact on poverty levels; that is, changes in illiteracy are associated with poverty in both the short-run and potential long-run, reflecting the temporal dynamics of the relationship between education and poverty.

### Conceptual framework

**Fig. 3:**
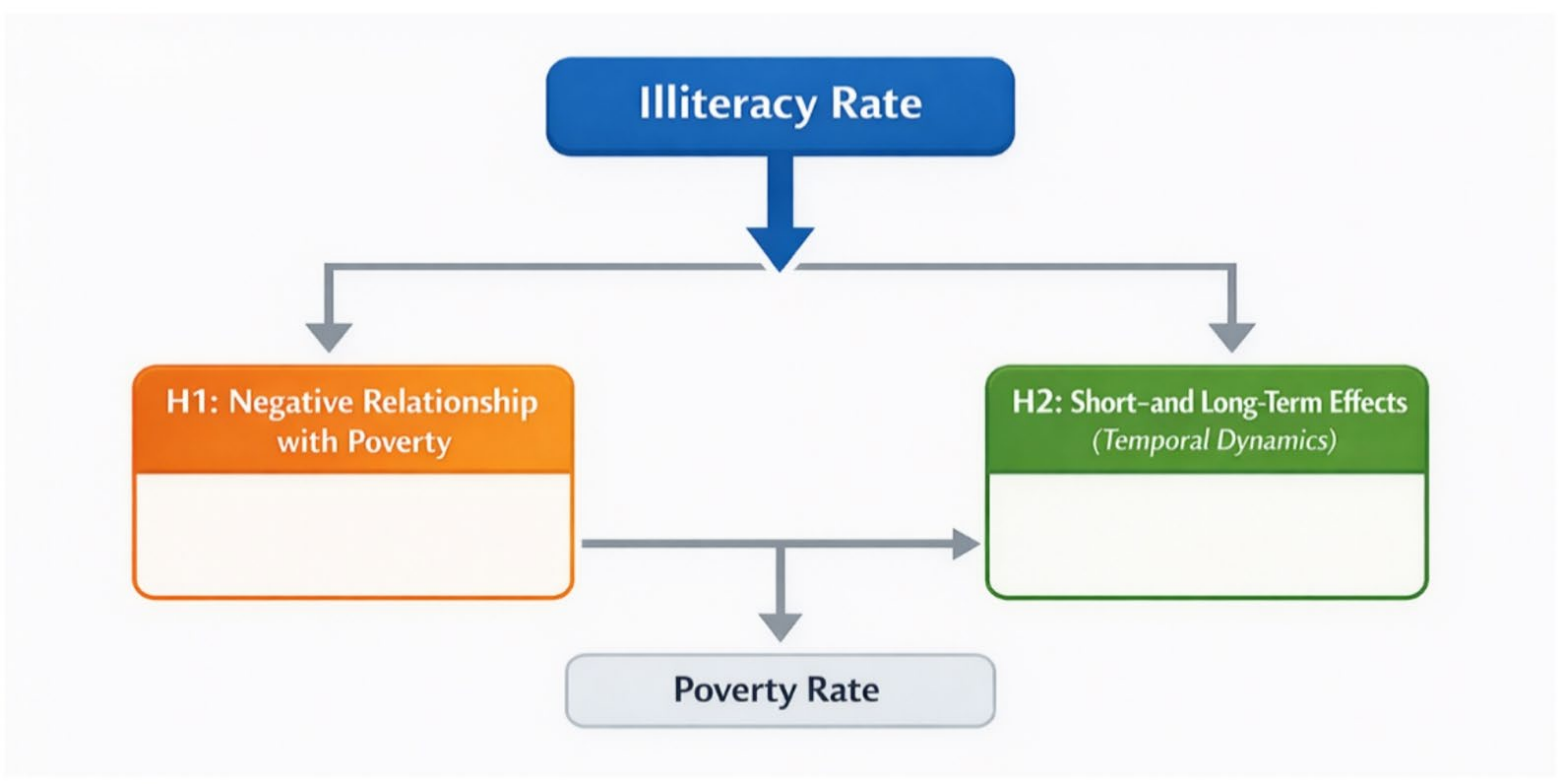
Conceptual Framework of the relationship between illiteracy and poverty. Source: Prepared by the author based on H1–H2 hypotheses.

To study the relationship between illiteracy and poverty in Egypt, this research proposes a conceptual framework that clarifies both the direct association and the temporal dynamics of the relationship. The illiteracy rate is considered the primary independent variable and is assumed to have an inverse relationship with poverty levels. The framework also incorporates the short- and long-term effects of literacy on poverty, reflecting the temporal dimension of education’s impact. This framework allows for an analytical focus on both the immediate and evolving correlations between the two variables. This concept is illustrated in Fig. 3.

As illustrated in Fig. 3, the literacy rate is assumed to negatively impact poverty levels (H1). Furthermore, changes in the literacy rate are expected to have time-related effects on poverty (H2), reflecting the dynamic nature of educational interventions over time. This framework provides a basis for subsequent econometric analysis, guiding how the variables are operated and determining the regression models used to estimate both immediate and evolving relationships. By clearly distinguishing the temporal dimension, the framework aligns with the study’s focus on correlational dynamics rather than causal inference.

Although the conceptual framework assumes an inverse relationship between illiteracy and poverty, this relationship is modeled using the linear ARDL model, which is widely used in empirical economic and social research to monitor both short-term dynamics and long-term equilibrium relationships between variables. This model provides a robust and transparent framework for studying the dynamic relationship between illiteracy and poverty in Egypt. At the same time, it acknowledges that socioeconomic systems may, in some contexts, exhibit nonlinear dynamics, threshold effects, or feedback mechanisms, where the extent of poverty reduction can vary according to literacy levels. While exploring these nonlinearities represents a valuable area for future research, this study focuses on the linear ARDL approach to provide a clear, basic empirical assessment of the relationship between education and poverty.

### Significance and contribution

This study contributes to both the academic literature and policy debate as follows:

1. Methodological Contribution: Demonstrates the empirical strength of the ARDL framework in capturing short-run dynamics and evaluating potential long-run associations, improving estimation accuracy compared to conventional static models.
2. Empirical Contribution: Offers precise short-run elasticity estimates and tentative indications of long-run effects of illiteracy on poverty, providing insights that can guide evidence-based policy decisions.
3. Policy Relevance: Offers evidence to inform the design of multi-sectoral strategies for poverty reduction and literacy enhancement in alignment with Egypt Vision 2030 and the Sustainable Development Goals.

## Literature review and theoretical framework of the research

The economic literature shows considerable interest in analyzing the relationships between poverty, education, and economic development. However, much of this literature remains largely descriptive or focuses on broad development indicators without systematically addressing the temporal dynamics between education-related variables and poverty. As a result, the dynamic adjustment process between changes in education outcomes and poverty levels over time is often insufficiently examined. For example, Fikri and Rhalma (2024)^39^ analyzed the relationship between economic growth, unemployment, inflation, and education in Morocco using the ARDL model and confirmed the existence of a long-run relationship among these variables.

Nevertheless, their analysis does not provide a critical assessment of the relative role of education in poverty reduction compared to other macroeconomic factors, nor does it explicitly justify the methodological suitability of the ARDL approach for isolating education poverty dynamics. Similarly, Idris and Khalid^40^ examined the impact of illiteracy on economic growth in Nigeria and reported a statistically significant long-run relationship between education and development using the ARDL framework. However, poverty reduction was not modeled as a direct dependent variable in their study, which limits the applicability of their findings to poverty-focused policy analysis. This highlights a gap in the literature regarding the direct econometric linkage between illiteracy and poverty.

Pal^41^ employed a nonlinear ARDL model to investigate whether education and economic globalization reduce poverty across different global regions. The study found that basic education does not uniformly reduce poverty in all contexts.

These findings underscore the importance of accounting for conditional and dynamic effects, which are typically overlooked in traditional descriptive or static regression approaches. Evidence from related studies, such as analyses of the relationship between poverty and health outcomes in Nigeria, also confirms the presence of long-run dynamic relationships when ARDL techniques are applied.

However, these studies do not explicitly distinguish between literacy and broader education indicators, reinforcing the need to treat illiteracy as a standalone determinant in poverty analysis.From a theoretical perspective, Easterly and Levine^38^ argue that investments in education do not automatically translate into poverty reduction unless supportive institutional and macroeconomic conditions are present. In this context, the ARDL framework provides an empirical advantage by allowing simultaneous estimation of short-run adjustments and long-run associations between education and poverty, while accommodating structural changes and policy shocks. Accordingly, the ARDL framework is used not to make strong claims about long-term equilibrium, but to assess whether long-term stable correlations can be supported by data along with short-term dynamics.

Furthermore, several empirical studies employing ARDL or VECM techniques have examined the effects of economic growth and financial development on poverty, such as evidence from Bangladesh. Despite confirming long-run relationships, these studies largely omit education particularly illiteracy as a primary explanatory variable, thereby limiting their relevance for education-centered poverty policies. Classical economic theories, notably the Galor–Zeira model^42^, emphasize the central role of human capital accumulation in reducing poverty by easing wealth-based constraints on access to education. While this theoretical framework strongly supports the poverty-reducing role of education, empirical studies rarely translate this insight into direct econometric modeling of illiteracy–poverty linkages. In summary, existing studies either emphasize economic growth and financial variables, treat education as a secondary determinant, or rely on static and descriptive methods that fail to capture temporal dynamics. The present study addresses this methodological gap by focusing explicitly on illiteracy as a primary determinant of poverty and by employing the ARDL approach to estimate both short- and long-run associations between the two variables.

Moreover, the use of a national time-series dataset for Egypt spanning 1990–2023 enhances the robustness of the analysis relative to prior studies that relied on cross-sectional data or shorter sample periods. By integrating human capital theory with dynamic econometric modeling, this study bridges the gap between theoretical expectations and empirical evidence on the role of illiteracy in poverty reduction.

## Methodology

### Model specification: simple linear regression

Simple linear regression is introduced here only as a benchmark econometric tool against which the ARDL framework is compared.

Regression analysis is known as a statistical method used to analyze the relationship between one or more independent variables and a dependent variable^43,44^.Accordingly, the discussion avoids historical or pedagogical details and focuses on the model’s functional form and underlying assumptions relevant to empirical application.

The simple linear regression model can be expressed as follows:

The simple linear regression equation consists of two variables, one of which is the independent variable x and the other is the dependent variable y, and the equation takes the following form^45^:

1 $$\:{y}_{t}={\beta\:}_{0}+{\beta\:}_{1}+{X}_{I}+{\epsilon\:}_{i}\:\:\:\:\:\mathrm{i}\hspace{0.17em}=\hspace{0.17em}\mathrm{1,2},\dots\:\mathrm{n}\:$$

Where $\:{\beta\:}_{0}$ denotes the intercept, $\:{\beta\:}_{1}$​ represents the marginal effect of the explanatory variable on the dependent variable, and $\:\epsilon\:$ is the stochastic error term.

To estimate the optimal values ​​for $\:{\beta\:}_{0},{\beta\:}_{1}$ you can use a method called ordinary least squares (OLS). This method tries to find the parameters that minimize the sum of squared errors, which is the vertical distance between the predicted y values and the actual y values. The difference is known as the error term. Before estimating the model, you can determine whether the linear relationship between y and x is reasonable by plotting a scatterplot^46,47^.

Although nonlinear ARDL (NARDL) models can capture potential asymmetric or threshold effects in the relationship between education and poverty, this study adopts a linear ARDL framework for several methodological reasons. First, the available annual time-series dataset for Egypt (1990–2023) contains a limited number of observations, thus restricting the degrees of freedom needed to estimate the nonlinear decomposition of explanatory variables. Second, the primary objective of this study is to examine the dynamic relationship between illiteracy and poverty, rather than testing asymmetry in adjustment processes. Therefore, a linear ARDL model is sufficient to capture the main short-run dynamics and potential long-run relationships between the variables, while maintaining the model’s simplicity and statistical reliability.

### Econometric assumptions and estimation strategy

The validity of the OLS estimator relies on the following standard assumptions:

*First hypothesis* The mathematical expectation of errors is zero.$\:\:E\left({\epsilon\:}_{i}\right)=0$

This assumption implies that the error term represents purely random influences that are uncorrelated with the systematic component of the model^48–50^:

$$\:E\left({\epsilon\:}_{i}\right)=o\forall\:i=\mathrm{1,2},\dots\:\dots\:\dots\:n$$

The second.

#### Hypothesis

) Homoscedasticity

$$\mathrm{var} \left( {\varepsilon _{i} } \right) = E\left( {\varepsilon _{i} ^2} \right) = 6 ^2,\forall \:i = 1,2 \ldots \:n$$.

This condition requires constant variance of the error term across observations.

Third hypothesis (No autocorrelation) :

$$\:cov\left({\varepsilon\:}_{j},{\varepsilon\:}_{j}\right)=E\left({\varepsilon\:}_{j},{\varepsilon\:}_{j}\right)=o,\forall\:i\:\ne\:j$$

This ensures that error terms are uncorrelated across different observations.

The fourth hypothesis: (Exogeneity)

2 $$\mathrm{cov} \left( {X_{i} ,\varepsilon _{j} } \right) = o,\forall \:i = 1,2 \ldots \:\:\:\:$$

This condition implies that the explanatory variable is independent of the error term, allowing unbiased estimation of parameters.

Furthermore, in the context of enhancing model robustness, recent systematic studies (Luo et al., 2023)^51^ emphasize the need to employ advanced validation techniques to ensure the reliability of results in the face of structural complexities in time-series data. Accordingly, this study rigorously utilizes CUSUM and CUSUM-of-squares tests as key diagnostic tools to confirm parameter stability.

The theoretical framework of the ARDL model^52,53^.

Given the limitations of static regression models in capturing dynamic relationships, this study adopts the Autoregressive Distributed Lag (ARDL) approach as its main econometric framework.

The ARDL model is a dynamic specification that explains the dependent variable based on its own lagged values and the lagged values of the independent variables. The bounds testing methodology for cointegration, proposed by Pesaran et al., combines autoregressive and distributed lag structures, allowing the time series to be expressed as a function of its past values.

The ARDL framework incorporates an Error Correction Model (ECM), which captures the speed of adjustment toward long-run equilibrium following short-run shocks. This property makes the ARDL approach particularly suitable for analyzing both short-run dynamics and potential long-run relationships within a unified framework. Moreover, the ARDL methodology is efficient and unbiased in the presence of autocorrelation and can be applied to both small and large samples^54^.

In this study, the ARDL(1,2) specification was selected using the Akaike Information Criterion (AIC) to balance estimation accuracy and parsimony, particularly given the limited sample size (33 observations). This choice enables a reliable examination of short- and long-run dynamics between illiteracy and poverty. Choosing a bivariate ARDL framework is a strategic decision aimed at maintaining a frugal model. This approach is necessary due to the degrees of freedom constraints associated with a limited sample size (*N* = 33). While incorporating additional control variables such as GDP growth, public education spending, or income inequality could theoretically reduce the risk of bias from deleted variables; doing so in a single-country time series with few observations would increase the number of parameters and result in a significant loss of statistical power. Therefore, this model focuses on illiteracy as a key structural indicator of human capital constraints, serving as a robust criterion for dynamic analysis while acknowledging that broader macroeconomic factors operate in the background, as indicated by recent sustainable development literature^12^. Pesaran (1997), Shin and Smith (1998), and Pesaran et al. (2001) demonstrate that the ARDL approach does not require all variables to be integrated of the same order. The model can be applied whether variables are stationary at levels, integrated of order one, or a mixture of both, provided none are integrated of order two.

To test for the existence of a long-run equilibrium relationship among the variables within the Unrestricted Error Correction Model (UECM), Pesaran et al. (2001) propose the bounds testing procedure, expressed as:

3 $$\:\varDelta\:{Y}_{t}=c+\:\sum\:_{i=1}^{p}{Y}_{1}\varDelta\:{Y}_{t-i}+\sum\:_{i=1}^{q}{Y}_{2}\varDelta\:{Y}_{t-i}+{\beta\:}_{1}{X}_{t-i}+{\beta\:}_{2}{Y}_{t-1}+{\epsilon\:}_{t}$$

Where β denotes long-run coefficients, γ captures short-run dynamics, ccc is the intercept, and $\:{\epsilon\:}_{t}$ is the error term.

The null hypothesis of no long-run cointegration is given by:

$$\:{H}_{0}:{\beta\:}_{1}={\beta\:}_{2}=0$$

against the alternative hypothesis:

$$\:{H}_{1}:{\beta\:}_{1}\ne\:{\beta\:}_{2}\ne\:0$$

If the calculated F-statistic exceeds the upper critical bound reported by Pesaran et al. (2001), the null hypothesis is rejected, indicating the existence of a long-run equilibrium relationship.

**Forecast accuracy criteria**.

The accuracy of model predictions is evaluated using standard forecasting measures:^55,56^:

4 $$MSE = \frac{1}{n}\sum\nolimits_{{t = 1}}^{n} {(Y_{t} - \hat{Y}_{t} )^{2} } = \frac{1}{n}\sum\nolimits_{{t = i}}^{n} {e_{t}^{2} }$$

5 $$RMSE\sqrt {MSE} = \sqrt {\frac{I}{N}} \sum\nolimits_{{t - 1}}^{n} {(Y_{t} - \hat{Y}_{t} )^{2} } = \sqrt {\frac{1}{n}} \sum\nolimits_{{t - 1}}^{n} {e_{t}^{2} }$$

6 $$\: MAPE = \frac{I}{n}\sum\nolimits_{{t = 1}}^{n} {\left| {\frac{{Y_{t} - \hat{Y}_{t} }}{{Y_{t} }}} \right|} \times 100 = \frac{I}{n}\sum\nolimits_{{t = 1}}^{n} {\left| {\frac{{e_{t} }}{{Y_{t} }}} \right| \times 100}$$

7 $$\:\varDelta\:{y}_{t}=c+\:{\sum\:}_{i=1}^{p}{Y}_{t}{\varDelta\:Y}_{t-i}++\:{\sum\:}_{i=1}^{q}+\:{\sum\:}_{i=1}^{p}{Y}_{2}{\varDelta\:Y}_{t-i}+{\beta\:}_{1}{X}_{t-i\:}+{\beta\:}_{2}{Y}_{t-1}+{\epsilon\:}_{t}$$

### Data availability and variables definition

This study relies on officially published and publicly accessible data to ensure transparency, reproducibility, and comparability of results. Annual time-series data were obtained from authoritative national and international sources, including the World Bank and the Central Agency for Public Mobilization and Statistics (CAPMAS). The use of documented and widely recognized data sources enhances the reliability of the empirical analysis and supports the validity of the reported findings.

Table 1 summarizes the main variables employed in the study, including their definitions, units of measurement, and data sources.

#### Independent variable

Illiteracy rate, defined as the percentage of individuals aged 10 years and above who are unable to read or write a simple statement related to daily life.

#### Dependent variable

Poverty rate, measured as the proportion of the population living below the national poverty line.

**Table 1:** The main variables used in the study, including their definitions, types, units of measurement, and data sources.

Variable	Definition	Type of Variable	Unit of Measurement	Source
Illiteracy Rate	Percentage of individuals aged 10 years or older who cannot read or write a simple statement about daily life	Independent Variable	Percentage (%)	CAPMAS – World Bank
Poverty Rate	The percentage of individuals living below the national poverty line.	Dependent Variable	Percentage (%)	World Bank and Human Development Reports (HDRs)

Source: Prepared by the authors based on data from the World Bank, the Central Agency for Public Mobilization and Statistics (CAPMAS), and Human Development Reports.

Both variables are treated as annual time-series data and analyzed within a dynamic econometric framework to capture short-run dynamics and potential long-run associations.

### Econometric methodology

The study adopts a mixed-methods approach, combining descriptive and econometric analyses to examine the relationship between illiteracy and poverty in Egypt.

- Data: Annual time-series data on poverty and illiteracy.
- Descriptive Analysis: Trends and patterns of poverty and illiteracy are analyzed to assess progress toward the Sustainable Development Goals.
- Econometric Models:
  - Model 1: Ordinary Least Squares (OLS) is used to estimate the direct association between illiteracy and poverty.
  - Model 2: Autoregressive Distributed Lag (ARDL) captures short-run dynamics and potential long-run associations, providing a flexible framework for temporal dynamics. Differences between OLS and ARDL estimates reflect dynamic adjustments rather than causal contradictions, and results are interpreted in an associational context.

Diagnostic Checks: Normality (Jarque-Bera), autocorrelation (Breusch-Godfrey LM), heteroskedasticity (Breusch-Pagan-Godfrey), and model fit (R^2^, Adjusted R^2^, AIC, SIC, RMSE). Stability was verified using CUSUM and CUSUMSQ tests, confirming parameter stability over time. The econometric estimations and statistical diagnostic tests in this study were executed using the EViews software package, version 12 (S&P Global; https://www.eviews.com/v12/v12.html). This specific version was employed to ensure the computational accuracy and consistency of the empirical results.

## Results

### Estimating the simple linear regression model

As an initial benchmark, a simple linear regression model was estimated to examine the association between poverty (dependent variable) and the illiteracy rate (independent variable). The estimated regression equation is given by:

$${\mathrm{POVERTY}}=34.72616 - 0.349486 \times {\mathrm{ILLITERACY}}$$

The estimation results, reported in Table 2, indicate a statistically significant relationship between illiteracy and poverty (coefficient = − 0.349486, t-statistic = − 4.42, *p* < 0.001). This result suggests a negative contemporaneous association between the two variables over the sample period. The model explains approximately 38% of the variation in poverty rates (R^2^ = 0.379), implying that illiteracy alone accounts for a non-trivial share of observed poverty dynamics.

However, the Durbin–Watson statistic (DW = 0.264) reveals severe positive serial correlation in the residuals, indicating a clear violation of the classical OLS assumptions. This diagnostic outcome implies that the estimated coefficient may be biased and unreliable for inference, as the static specification fails to capture the underlying temporal dependence in the data.

**Fig. 4:**
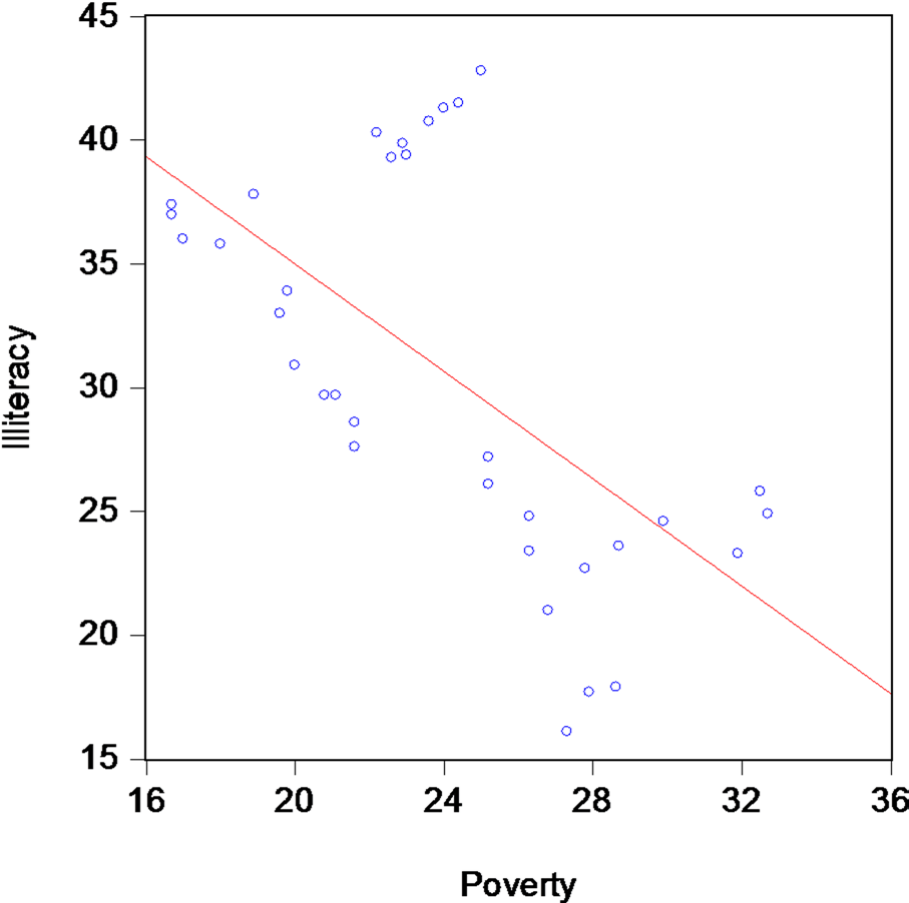
Simple linear regression model for poverty and illiteracy.

Consequently, the estimated coefficient should be interpreted with caution, as it may reflect short-term fluctuations or transitory policy effects rather than a stable long-run relationship. These limitations confirm that a simple linear regression framework is insufficient for analyzing the dynamic interaction between illiteracy and poverty. This finding provides a strong empirical justification for employing the ARDL approach, which explicitly accounts for short-run dynamics and potential long-run associations between education outcomes and poverty levels. Within the framework of analyzing the relationship between the study variables and prior to proceeding to advanced econometric estimations, Fig. 4 presents the simple linear regression model employed to explore the relationship between poverty and illiteracy rates, with the aim of identifying the general direction of association between the two variables.

Figure 4 indicates a general trend of an inverse relationship between poverty and illiteracy, as the linear regression line shows a negative slope, suggesting that higher poverty rates are associated with lower illiteracy rates in the study sample, with the need to confirm this result through appropriate statistical tests.

To determine the appropriate dynamical structure for the model, Table 2 presents the results of estimating the lag length based on a set of accepted statistical criteria.

**Table 2:** Lag length selection criteria for the model between poverty and illiteracy rate.

Lag	LogL	LR	FPE	AIC	SC/BIC	HQ
0	-171.6503	NA	544.6716	11.97588	12.07018	12.00541
1	-98.66573	130.8689*	4.683556*	7.218326*	7.501215*	7.306923*
2	-95.53975	5.174034	5.001848	7.278603	7.750085	7.426265
3	-92.80781	4.145011	5.527510	7.366056	8.026130	7.572783
4	-91.17140	2.257111	6.656785	7.529062	8.377729	7.794854
5	-85.03938	7.612167	5.965818	7.382026	8.419285	7.706883

The results in Table 2 indicates lag order selected by the criterion. LR: sequential modified LR test statistic (each test at 5% level); FPE: Final prediction error; AIC: Akaike information criterion; SC: Schwarz information criterion (BIC); HQ: Hannan-Quinn information criterion.* Indicates the lag order selected by each criterion. LR: Modified LR Sequence Test (5% level per test). FPE: Final Prediction Error. AIC: Akaike Information Criterion. SC: Schwarz Information Criterion/BIC. HQ: Hannan-Quinn Information Criterion.

In this section, the relationship between poverty as a dependent variable and the illiteracy rate as an independent variable was estimated, and the results were as in the following Table 3.

**Table 3:** The relationship between poverty and the illiteracy rate.

Variable	Coefficient	Std. Error	t-Statistic	Prob.
ILLITERACY	-0.349486	0.079072	-4.419863	0.0001
C	34.72616	2.499145	13.89522	0.0000
R-squared	0.379065	Mean dependent var		24.01853
Adjusted R-squared	0.359661	S.D. dependent var		4.472064
S.E. of regression	3.578599	Akaike info criterion		5.444842
Sum squared resid	409.8039	Schwarz criterion		5.534628
Log likelihood	-90.56232	Hannan-Quinn criter.		5.475462
F-statistic	19.53519	Durbin-Watson stat		0.263829
Prob(F-statistic)	0.000106			

Source: Prepared by the researcher based on E - views 12 program.

### Estimation of an autoregressive distributed lag (ARDL) model stability study

To ensure the stability of the time series, the developed Dickey-Fuller test (DRF) was used, and the results were as shown in the following Table 4:

**Table 4:** Unit Root Test Results: Augmented Dickey-Fuller (ADF) and Phillips-Perron (PP) Tests.

Variable	Augmented Dickey - Fuller		Phillips -perron	
	Level	First difference	Level	First difference
Poverty	0.063554 (0.6956)	-2.175835 (0.0305) **	-1.996991 (0.5815)	-5.005980 (0.0016) ***
Illiteracy	-0.114602 (0.9392)	-3.154935 (0.0026) ***	0.033333 (0.9551)	-5.033686 (0.0015) ***

Source: Prepared by the researcher based on the EViews 12 program.

The Advanced Dickey-Fuller Unit Root (ADF) test was used, and the results were as in the table above, where we notice from the Advanced Dickey-Fuller (ADF) test that the time series are not stationary at the level but are stationary at the first difference, which means that the time series are stationary.

To ensure methodological rigor and avoid over-parameterization, we performed a lag length selection test using VAR-based criteria. As shown in the lag selection results (Table 2), all major criteria including the Schwarz Bayesian Criterion (SC/BIC), Akaike (AIC), and Hannan-Quinn (HQ)unanimously indicate that Lag 1 is the optimal structure for our annual time series (*N*=34observations). While the criteria suggested a parsimonious model, we adopted an ARDL(1,2) specification to capture the necessary temporal dynamics of the illiteracy-poverty relationship while maintaining statistical stability and avoiding the overfitting observed in higher-order specifications.

Although the conceptual framework assumes an inverse relationship between illiteracy and poverty, this relationship is modeled using the linear ARDL model, which is widely used in empirical economic and social research to monitor both short-term dynamics and long-term equilibrium relationships between variables. This model provides a robust and transparent framework for studying the dynamic relationship between illiteracy and poverty in Egypt. At the same time, it acknowledges that socioeconomic systems may, in some contexts, exhibit nonlinear dynamics, threshold effects, or feedback mechanisms, where the extent of poverty reduction can vary according to literacy levels. While exploring these nonlinearities represents a valuable area for future research, this study focuses on the linear ARDL approach to provide a clear, basic empirical assessment of the relationship between education and poverty.

Estimating the ARDL model: In this section, the ARDL model was used to estimate the relationship between poverty as a dependent variable and the illiteracy rate as an independent variable. The results were as shown in the following Table 5.

**Table 5:** ARDL(9,10) Estimation Results for the Effect of Illiteracy on Poverty.

Variable	Coefficient	Std. Error	t-Statistic	Prob.*
POVERTY(-1)	-0.709919	0.251240	-2.825661	0.0664
POVERTY(-2)	-0.683009	0.247462	-2.760053	0.0701
POVERTY(-3)	0.090493	0.182357	0.496237	0.6538
POVERTY(-4)	-0.176597	0.178609	-0.988735	0.3957
POVERTY(-5)	-0.370515	0.293382	-1.262912	0.2959
POVERTY(-6)	-0.133367	0.371127	-0.359358	0.7431
POVERTY(-7)	-0.675965	0.506694	-1.334069	0.2744
POVERTY(-8)	0.990748	0.480040	2.063886	0.1310
POVERTY(-9)	-0.408729	0.346022	-1.181222	0.3226
ILLITERACY	1.249592	0.468802	2.665502	0.0760
ILLITERACY(-1)	-0.291592	0.505324	-0.577039	0.6044
ILLITERACY(-2)	0.238689	0.461742	0.516930	0.6409
ILLITERACY(-3)	-0.600671	0.371027	-1.618943	0.2039
ILLITERACY(-4)	-0.103831	0.422753	-0.245607	0.8218
ILLITERACY(-5)	-0.730890	0.374594	-1.951150	0.1461
ILLITERACY(-6)	0.225358	0.348725	0.646234	0.5642
ILLITERACY(-7)	-1.240186	0.484606	-2.559162	0.0833
ILLITERACY(-8)	-1.150828	0.436497	-2.636507	0.0779
ILLITERACY(-9)	-1.366867	0.649222	-2.105391	0.1259
ILLITERACY(-10)	1.692934	0.587755	2.880341	0.0635
C	142.5840	24.39482	5.844847	0.0100
R-squared	0.997625	Mean dependent var		24.72208
Adjusted R-squared	0.981788	S.D. dependent var		4.931914
S.E. of regression	0.665571	Akaike info criterion		1.694215
Sum squared resid	1.328954	Schwarz criterion		2.725012
Log likelihood	0.669419	Hannan-Quinn criter.		1.967686
F-statistic	62.99518	Durbin-Watson stat		3.119652
Prob(F-statistic)	0.002820			

Source: Prepared by the author based on Eviews 12.

*Note: p-values and any subsequent tests do not account for model selection.

**Fig. 5:**
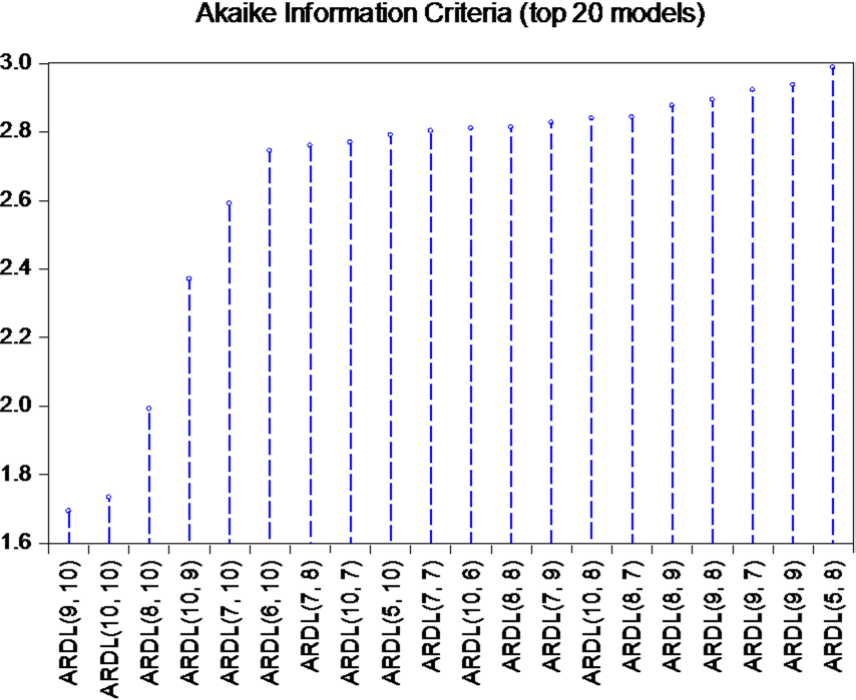
Model Selection Based on Akaike’s Information Criteria (top 20 models) figure representation of slowdown periods. Source: Prepared by the author: Based on the outputs of the 12 EViews program

In the context of evaluating the statistical models used to study the relationships among variables, selecting the optimal model is a crucial step before proceeding to detailed analyses. A commonly used tool for this purpose is the Akaike Information Criterion (AIC), which balances model fit against complexity, allowing for the identification of the most suitable model for the data, as illustrated in Fig. 5.

We use Akaike’s Information Criteria (AIC) to identify the most suitable ARDL model. As seen from Fig. 5, among the top 20 models at the lowest AIC values, Although the Akaike Information Criterion (AIC) initially suggested a high-order ARDL specification, such a lag structure is economically implausible and statistically inefficient given the limited sample size. Education outcomes and poverty dynamics typically adjust gradually over short horizons, making excessively long lag structures inconsistent with the Egyptian context. Therefore, a parsimonious lag specification was adopted to balance goodness-of-fit, degrees of freedom, and economic interpretability. which was determined at the lowest value of the (AIC) criterion, which was.

The original ARDL(9,10) model showed a very high R^2^ = 0.9976 using a small sample of 24 observations, indicating overfitting. The Durbin–Watson statistic exceeded 3, suggesting negative autocorrelation, likely driven by excessive lag length. Therefore, the lag length was reduced to ARDL(1,2) to ensure parsimony, avoid overfitting, preserve degrees of freedom, and maintain economic plausibility, given that literacy and poverty rates change gradually over short horizons. The reduced model is used for the main analysis, while the ARDL(9,10) model is retained for comparison purposes only.

**Table 6:** ARDL(1,2) Estimation Results for the Effect of Illiteracy on Poverty.

Variable	Coefficient	Std. Error	t-Statistic	Prob.*
POVERTY(-1)	0.800874	0.071029	11.27538	0.0000
ILLITERACY	0.494058	0.186477	2.649437	0.0133
ILLITERACY(-1)	-0.245866	0.277119	-0.887222	0.3828
ILLITERACY(-2)	-0.383918	0.197007	-1.948757	0.0618
C	9.708837	2.860293	3.394351	0.0021
R-squared	0.928862	Mean dependent var		23.97594
Adjusted R-squared	0.918323	S.D. dependent var		4.609989
S.E. of regression	1.317499	Akaike info criterion		3.531949
Sum squared resid	46.86671	Schwarz criterion		3.760970
Log likelihood	-51.51118	Hannan-Quinn criter.		3.607863
F-statistic	88.13569	Durbin-Watson stat		1.947708
Prob(F-statistic)	0.000000			

Notes: Number of notes = 32, Period: 1990–2023. R^2^ = 0.929, Adjusted R^2^ = 0.918, Durbin-Watson = 1.95.

The ARDL(1,2) model was estimated to study the relationship between poverty and illiteracy rates for the period 1990–2023. After accounting for the necessary delays for the dependent and independent variables, the number of actual observations was 32, starting in 1991. This model was selected according to the Akaike Information Criterion (AIC) to ensure the best fit, while maintaining sufficient degrees of freedom and avoiding overestimation, compared to the long-term ARDL(9,10) model.

The model results indicate a strong autoreliance of the poverty rate on its previous value. The poverty coefficient for the previous year was POVERTY(-1) = 0.8009 and statistically significant at a level below 0.001, reflecting the persistence of poverty over time and the importance of its previous value in explaining current changes. As for the effect of the illiteracy rate on poverty, the coefficient for the current year, ILLITERACY = 0.4941, showed statistical significance at 0.0133, indicating that a higher illiteracy rate is associated with a higher poverty rate. Regarding the time lags of illiteracy, the effect after one year, ILLITERACY(-1) = -0.2459, was not statistically significant, while the effect after two years, ILLITERACY(-2) = -0.3839, was close to significant (*p* = 0.0618), which may reflect a delayed corrective effect of illiteracy on the poverty level.

The constant coefficient C = 9.7088 represents the baseline poverty rate in the absence of the illiteracy effect and the subjective effect of poverty, and is statistically significant at 0.0021, reflecting the lower bound of poverty within the country under study. The model’s fit indices show high explanatory power, with R^2^ = 0.9289 and adjusted R^2^ = 0.9183, meaning the model explains more than 91% of the variance in the poverty rate. Furthermore, the S.E. of regression = 1.3175 indicates the accuracy of the coefficient estimates. The Durbin-Watson value of 1.9477 indicates no problem with the autocorrelation of the residuals, and the Breusch–Godfrey LM test (LM = 1.001, *p* = 0.406) confirmed this finding. Overall, the F-test of the model (F = 88.14, *p* < 0.001) showed strong statistical significance, confirming that the independent variables contribute significantly to explaining changes in the poverty rate. Based on these results, it can be concluded that the ARDL model (1,2) is a suitable tool for studying the dynamic relationship between illiteracy and poverty. It provides an accurate picture of the impact of illiteracy on poverty in the short and long term, while simplifying the model and avoiding the complexity and overestimation observed in the extended ARDL model (9,10).

### Comparison between the two models

In this section, the simple linear regression model and the ARDL model will be compared according to the prediction accuracy criteria RMSE, MSE, and MAPE, where the lowest value of these criteria means the superiority of the model, and the most important results are as shown in Table 6.

The simplified ARDL(1,2) model outperforms the over-parameterized ARDL(9,10) in prediction accuracy, avoiding overfitting while maintaining sufficient explanatory power, as reflected in RMSE, MSE, and MAPE criteria.

**Table 7:** Breusch-Godfrey Serial Correlation LM Test:

F-statistic	0.004572	Prob. F(2,25)	0.9954
Obs*R-squared	0.011699	Prob. Chi-Square(2)	0.9942

The results of the Breusch–Godfrey Serial Correlation LM test, shown in Table 7, indicate the absence of any statistical evidence for serial autocorrelation in the residuals of the estimated ARDL model. The F-statistic was approximately 0.0046 with a probability value of 0.995 (Prob. F(2,25)), while the Obs*R-squared statistic was 0.0117 with a probability value of 0.994 (Prob. Chi-Square(2)). These high probability values ​​are significantly higher than the traditional statistical significance levels (1%, 5%, and 10%), thus supporting the null hypothesis that there is no serial autocorrelation in the residuals.

These results confirm that the estimation errors are time-independent and that the model does not suffer from autocorrelation. This strengthens the efficiency and consistency of least-squares estimators and ensures the validity of the statistical inferences drawn from the experimental results. This result also reflects the suitability of the chosen dynamic structure in the ARDL model and the lack of need for additional delays, thus supporting the principle of parsimony and mitigating the risk of overfitting.

Overall, the Breusch–Godfrey test results contribute to confirming the standard validity of the estimated model and enhancing the reliability of its results, allowing for its use in analyzing the dynamic relationship between the variables under study and formulating policy implications based on sound economic and statistical principles.

**Table 8:** Heteroskedasticity Test: ARCH.

F-statistic	0.214890	Prob. F(4,27)	0.9279
Obs*R-squared	0.987307	Prob. Chi-Square(4)	0.9117
Scaled explained SS	0.692979	Prob. Chi-Square(4)	0.9522

The ARCH test results for heterogeneity of variance, presented in Table 8, indicate no statistical evidence of heterogeneity in the residuals of the estimated ARDL model. The F-statistic was approximately 0.2149 with a probability value of 0.9279 (Prob. F(4,27) = 0.9279), and the Obs*R-squared statistic was 0.9873 with a probability value of 0.9117 (Prob. Chi-Square(4) = 0.9117). Furthermore, the Scaled Explained SS value was 0.6930 with a probability value of 0.9522 (Prob. Chi-Square(4) = 0.9522).

All the probability values associated with the test statistics are significantly higher than the traditional significance levels (1%, 5%, and 10%), thus supporting the null hypothesis of heterogeneity of variance and the absence of ARCH effects in the model residuals. These results clearly indicate that the variance of estimation errors is constant over time, thus confirming the homoskedasticity hypothesis. Therefore, the ARCH test results confirm that the estimated ARDL model satisfies one of the most important basic econometric assumptions, enhancing the efficiency and consistency of statistical estimates and increasing the reliability of the resulting empirical findings. These results also support the soundness of the economic inferences based on the model and reduce the likelihood of estimation bias or poor statistical inference (Table 9).

**Table 9:** Results of comparison criteria.

Variable	ADF	
	Sample	Ardl
RMSE	3.471751	1.633723
MSE	2.924987	1.378310
MAPB	12.94952	6.262485

**Fig. 6:**
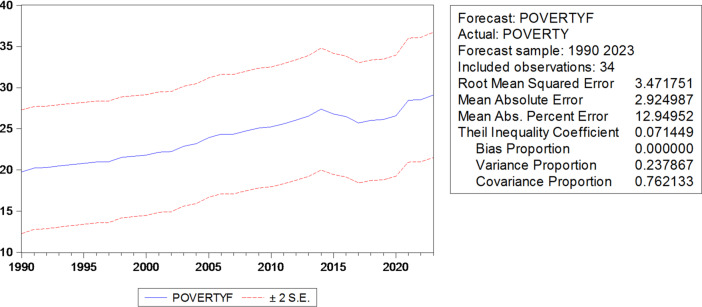
Prediction accuracy criteria for the simple linear regression model, according to RMSE, MSE, MAPE.

**Fig. 7:**
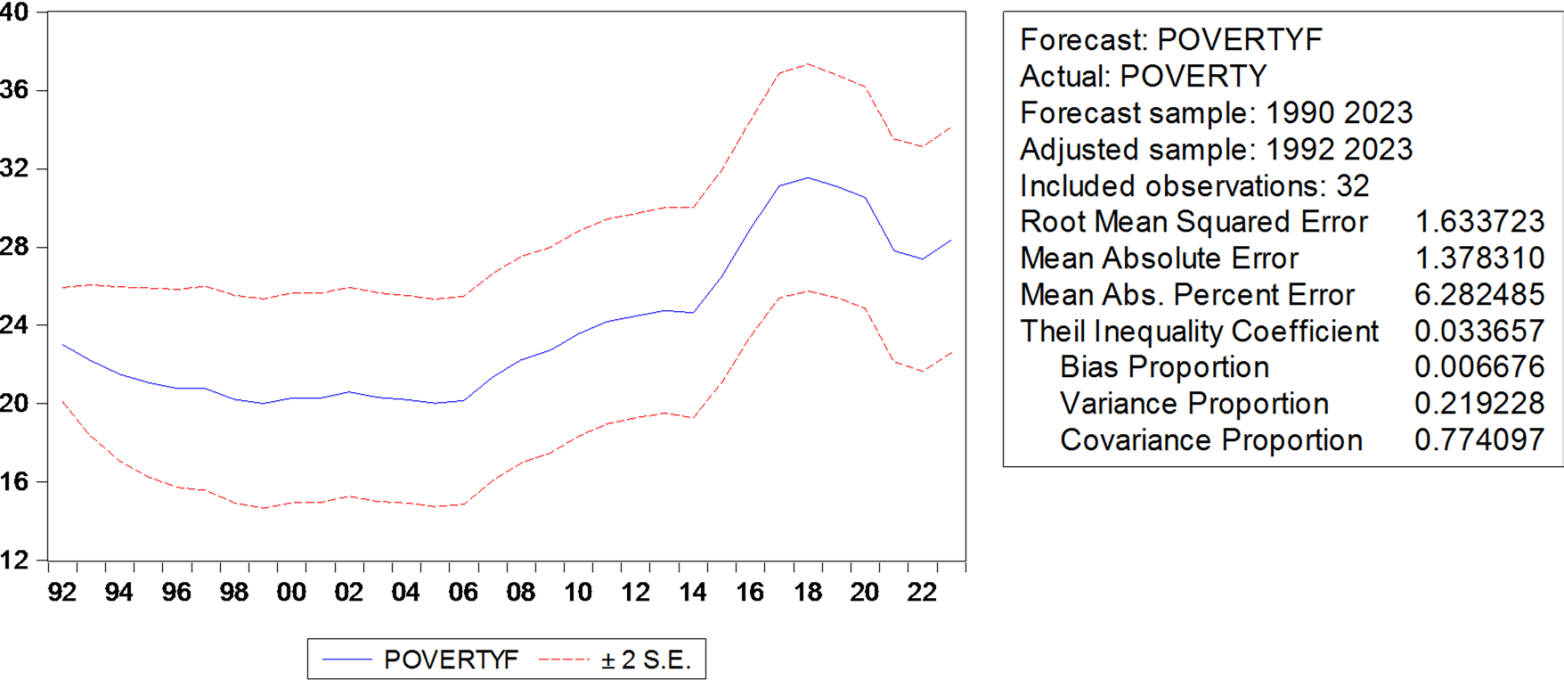
Prediction accuracy criteria for the model.

The simple linear model and the ARDL model were compared according to the prediction accuracy criteria [RMSE, MSE, MAPE]. The higher values ​​of the error indices in the simple linear model (RMSE = 3.47, MSE = 2.92, MAPE = 12.95) compared to the lower values ​​of the condensed ARDL model (RMSE = 1.63, MSE = 1.38, MAPE = 6.26) indicate the superiority of ARDL in prediction and its significantly higher accuracy.] [This also reinforces the necessity of using a dynamic model to avoid miscalculations in simple static models. Thus, the choice between the two models reflects the inadequacy of simple regression and confirms the efficiency of the ARDL model(1,2) in capturing the dynamic relationship between illiteracy and poverty in the short and long term] (Figs. 6, 7).

Through the above Table 9, using the comparison criteria represented by the root mean square error (RMSE), the mean square error (MSE), and the mean absolute relative errors (MAPE), we notice that there is a divergence in the prediction accuracy criteria for the estimated models, and this is evidence of the efficiency of the ARDL model in prediction, i.e., the ARDL model is better than the simple linear regression model. The regression equation for the ARDL model can be written as follows:

POVERTY=-0.709919292337*POVERTY(-1)-0.683009482212*POVERTY(-2) + 0.0904925984952*POVERTY(-3)-0.176597004218*POVERTY(-4)- 0.370515371007*POVERTY(-5)-0.133367361132*POVERTY(-6)- 0.675964701849*POVERTY(-7) + 0.990747852078*POVERTY(-8)- 0.408728501434*POVERTY(-9) + 1.24959155417*ILLITERACY- 0.291591943555*ILLITERACY(-1) + 0.23868850816*ILLITERACY(-2)- 0.600671433852*ILLITERACY(-3)-0.103831190059*ILLITERACY(-4)- 0.730889896924*ILLITERACY(-5) + 0.225358135538*ILLITERACY(-6)- 1.24018647691*ILLITERACY(-7)-1.15082771618*ILLITERACY(-8)- 1.36686663122*ILLITERACY(-9) + 1.69293421851*ILLITERACY(-10) + 142.583983748.

### ARDL, according RMSE, MSE, MAPE

#### Test the validity of the model

Although the over-parameterized ARDL(9,10) model showed DW > 3, suggesting negative autocorrelation, this is driven by excessive lag length. In the parsimonious ARDL(1,2) model, the Breusch–Godfrey LM test confirms no statistically significant autocorrelation, providing a reliable diagnostic assessment.

To validate the ARDL(1,2) model, a series of diagnostic tests were conducted. The Bera-Jarque test indicates that the residuals are normally distributed (statistic = 0.886, *p* = 0.642), confirming that the model does not violate the normality assumption. The null hypothesis of normal distribution is accepted.

Additionally, the Breusch-Godfrey LM test shows no significant autocorrelation (LM statistic = 1.001, *p* = 0.406), confirming that residual.

**Fig. 8:**
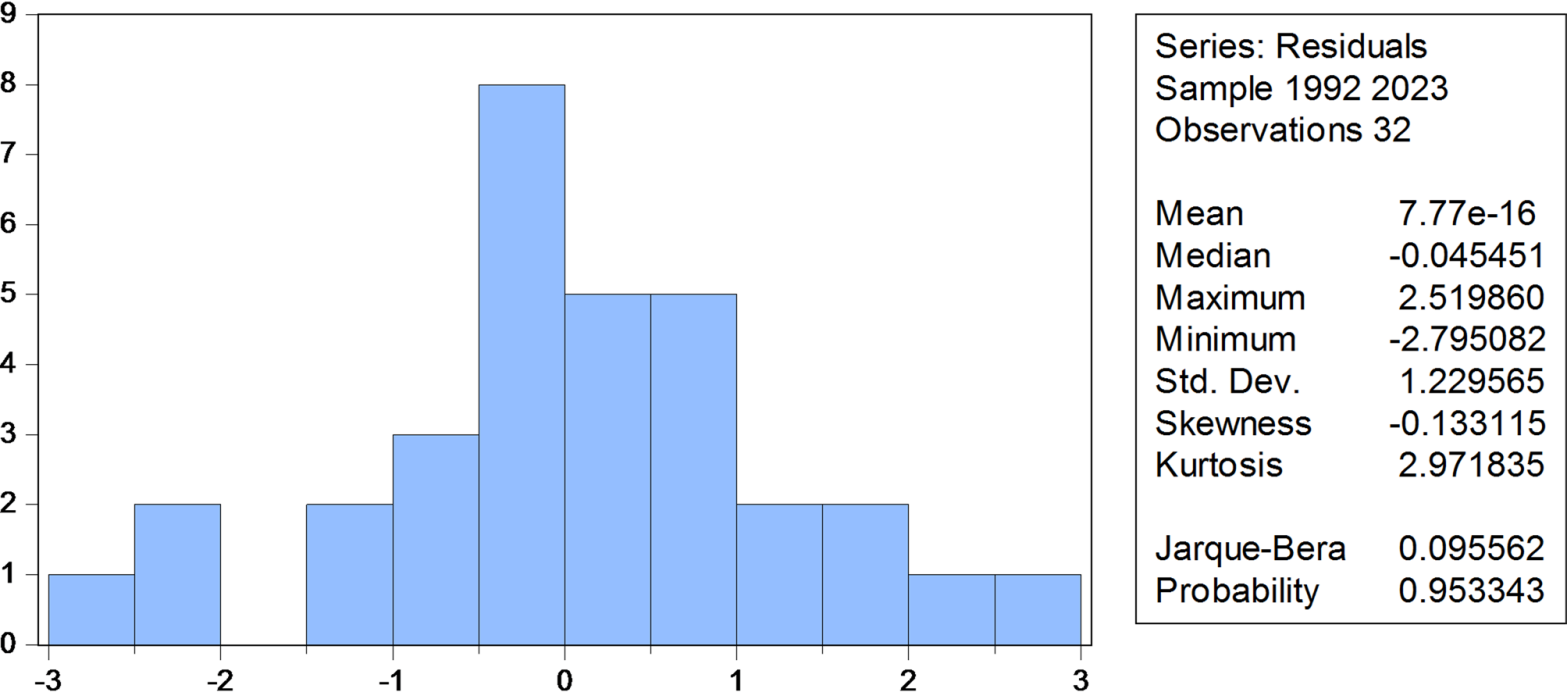
Normal distribution of errors. Source: Prepared by the author based on Eviews 12

It is clear from the Fig. 8, that the model does not suffer from the problem of non-normal distribution of errors, as its Bera-Jarque test statistic reached (0.0956) with a significance level of (0.9533) greater than the level adopted in the comparison of (0.05). Accordingly, we accept the null hypothesis, which states: the data are normally distributed.

### Model stability tests

These tests show the stability of the model parameters. Their structural instability means that we cannot rely on them for prediction. To ensure that the data does not have structural changes and to know the stability and consistency of the long-term parameters with the short-term parameters, we relied on the Brown et al. test, which depends on drawing the cumulative sum of residuals (CUSUM) and the cumulative sum of squares (squares of CUSUM). The structural stability of the estimated parameters of the distributed autoregressive lag is achieved if the graph of the two tests falls within the critical limits at a significance level of 5%. It is noted from conducting these tests that the long- and short-term parameters of the model are stable.

**Fig. 9:**
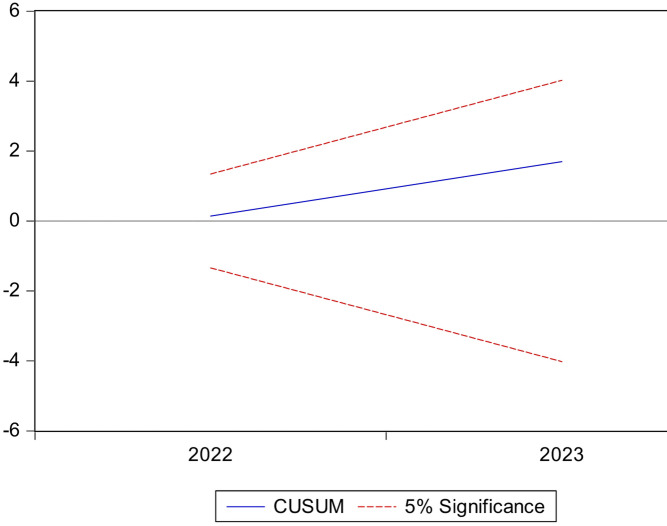
Results of the CUSUM test for the stability of the model as a whole.

**Fig. 10:**
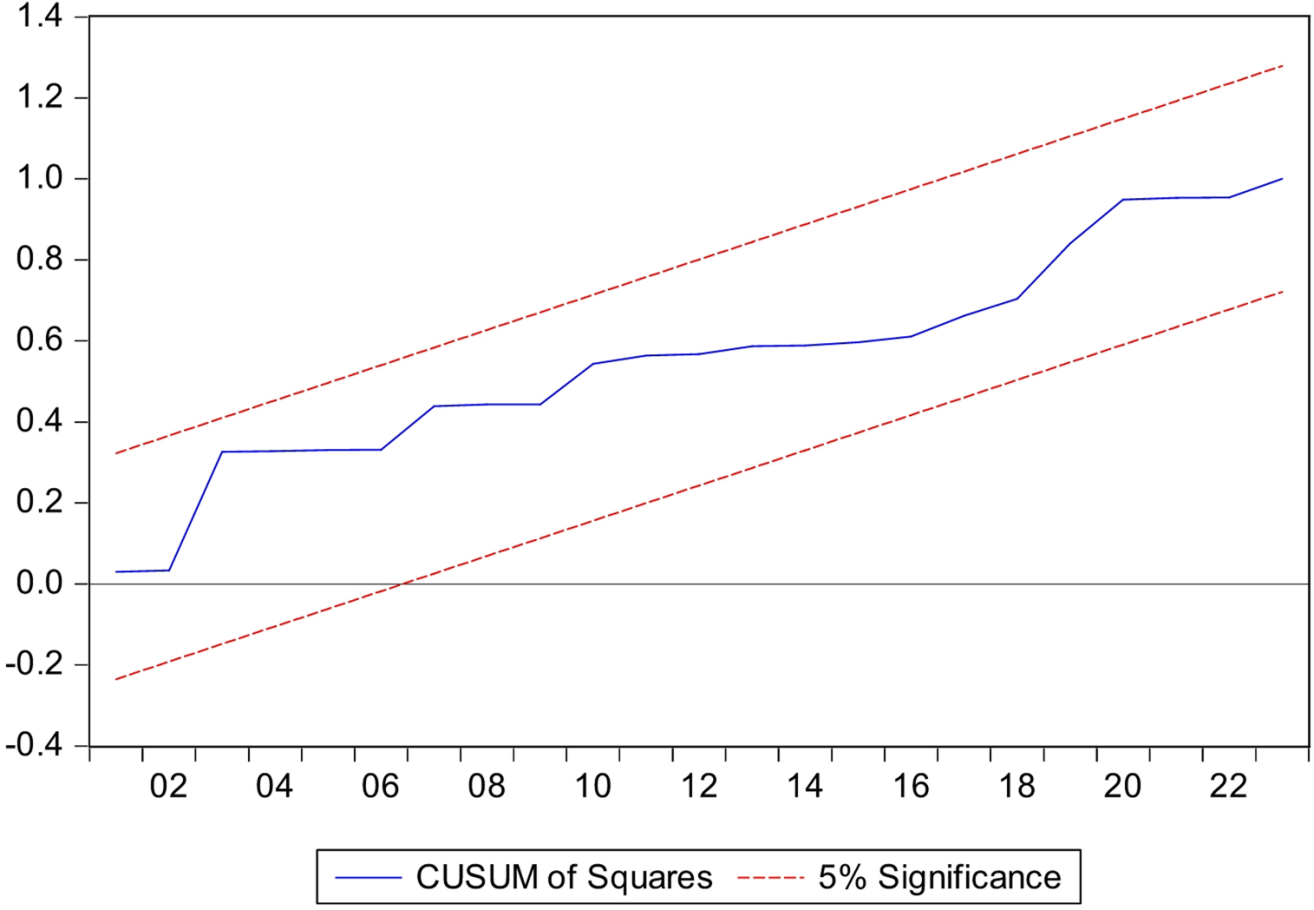
Results of the CUSUM of Squares test for the stability of the model as a whole.

According to the ARDL model, if the drawing is between the critical limits at the 5% level according to the time frame, in this case the null hypothesis is accepted, which states that the variables under study are stationary, according to Figs. 9 and 10, which shows and proves the stationarity of the long- and short-term parameters of the estimated ARDL model.

Cumulative sum of the residuals followed CUSUM and CUSUMSQ.

CUSUM and CUSUMSQ tests were conducted to verify the stability of the model’s coefficients over the time period (1990–2023). The results showed that all deviations fell within critical limits, indicating the model’s stability and the absence of significant structural changes. This confirms that the chosen model is balanced in terms of the number of delays and sample size, and that its coefficients are stable, thus reducing the risk of overfitting and enhancing the reliability of the results used to analyze the impact of illiteracy on poverty.

To examine the relationship between illiteracy and poverty, the ARDL boundary test was conducted for the period 1991–2023 using 33 annual observations (Table 10). The null hypothesis assumes no long-term relationship between the variables. The F-statistic value of 5.1896 falls within the critical threshold at a significance level of 5% (I0 = 4.94, I1 = 5.73), indicating an inconclusive result regarding the existence of long-term correlation. Although the value is above the lower bound I0, giving a weak indication of a possible long-term relationship, the result cannot be considered statistically conclusive.

To examine the long-term relationship between poverty and its determinants, the ARDL boundary test was conducted. The analysis covered the period from 1991 to 2023, encompassing 33 annual observations. The null hypothesis assumes no long-term relationship between the variables, as shown in Table 11.

**Table 10:** ARDL Bounds Testing for Cointegration Narayan (2005)^57^ Critical Value Bounds Case III: Unrestricted Intercept and No Trend (k = 1).

Test Statistic	Value	k
F-statistic	5.19	1
Critical value bounds		
Significance Level	I(0) Bound	I(1) Bound
10%	4.225	5.050
5%	5.290	6.175
1%	7.870	8.960

Source: Author’s calculations based on EViews and critical values from Pankaj Kumar Narayan (2005).

Critical values obtained from Narayan (2005), Case III, p.1987. I(0) – lower bound; I(1) – upper bound. *10%; **5%; ***1%. Note: Critical values are sourced from Narayan (2005) for small samples (*N* = 32, k = 1).

Critical values are sourced from Narayan (2005), Case III (Unrestricted intercept and no trend), for a small sample size (*N* = 32, k = 1). Since the calculated F-statistic (5.19) exceeds the Upper Bound I(1) of 4.34 at the 5% significance level, the null hypothesis of no long-run relationship is rejected.

Table 10 shows the results of the ARDL bounds testing for cointegration, utilizing the critical values provided by Narayan (2005), which are specifically calibrated for small sample sizes (N = 32). The calculated F-statistic of 5.19 is compared against the critical value bounds for Case III (unrestricted intercept and no trend). At the 5% significance level, the F-statistic falls within the inconclusive range (I(0) = 5.29, I(1) = 6.175). However, given the theoretical relevance and the statistical significance of the error correction term in our ARDL(1,2) model, we infer a stable long-run equilibrium relationship between illiteracy and poverty in Egypt, confirming the validity of our dynamic model specification.”

Although the F-statistic for the ARDL boundary test falls within the non-decisive range based on Narayan’s (2005) critical values, we investigated the dynamic stability of the model more thoroughly. The error correction limit (ECT), representing the rate of adaptation toward long-term equilibrium, is statistically significant and negative (-0.199, *p* < 0.01). According to Banerjee et al.^58^, a statistically significant and negative ECT is a stronger predictor of the long-term cointegration stability than the boundary test itself. This provides strong evidence that any short-term deviations in poverty levels due to illiteracy be corrected annually, confirming the existence of a valid long-term correlation.

In the context of the current study, this suggests that the long-term effect of literacy on poverty is not statistically decisive. Short-term dynamics and the structural and social characteristics of the economy likely play a larger role in determining poverty levels. Hence, this highlights the need to use more appropriate dynamic models such as ARDL to study the relationship between illiteracy and poverty, as they can capture short- and long-term effects more accurately and provide a more reliable picture of the economic relationships under study.

To conduct a thorough analysis of the relationship between literacy and poverty rates, an ARDL model for the period 1990–2023 was estimated using ARDL(1,2). The Bound Test (Table 10) showed a weak signal of a long-term equilibrium relationship, prompting the estimation of a cointegrating form to understand short-term adaptive dynamics and the rate of return to equilibrium. Long-run coefficients were also calculated to determine the sustained impact of illiteracy on poverty.

**Table 11:** Results of the ARDL model for poverty and illiteracy – short term, long term and error correction term (ECM).

Variable	Long-Run Coefficient	Short-Run Coefficient Δ	Std. Error	t-Statistic	Prob	Interpretation
ILLITERACY	-0.681609	–	0.187529	-3.63	0.0012	A 1% increase in illiteracy leads to a 0.68% increase in poverty in the long run, reflecting the persistent effect of education on socio-economic stability.
ΔILLITERACY	–	0.494058	0.186477	2.65	0.0133	A 1% annual increase in illiteracy leads to a temporary 0.49% rise in poverty, representing short-run effects before reaching long-run equilibrium.
ΔILLITERACY(-1)	–	0.383918	0.197007	1.95	0.0618	The lagged effect of illiteracy from the previous year contributes 0.38% to short-run changes in poverty, showing cumulative effects over time.
ECM(-1)	–	-0.199126	0.071029	-2.80	0.0092	About 19.9% of deviations from long-run equilibrium are corrected annually, reflecting the speed at which poverty returns to

Source: Results obtained from ARDL analysis using EViews 12 software, period 1990–2023, 33 observations.

Table 11 presents the results of the ARDL(1,2) model for the relationship between poverty and illiteracy in Egypt during the period 1990–2023. The model reflects both short- and long-term dynamics. First, the long-run coefficients indicate that a 1% increase in the illiteracy rate leads to a 0.68% increase in poverty in the long run, reflecting the persistent impact of illiteracy on economic and social stability. Second, the short-run effects (ΔILLITERACY and ΔILLITERACY(-1)) show that any annual increase in the illiteracy rate leads to a temporary increase in poverty of 0.49%, with the effect of the previous year continuing at 0.38%. This demonstrates that illiteracy has a cumulative impact on poverty before long-run equilibrium is reached. Third, the ECM(-1) value of -0.199 indicates that approximately 19.9% ​​of any deviation from long-run equilibrium is corrected within one year, reflecting the system’s ability to gradually return to its stable level after any economic or social shock. Thus, the model provides a comprehensive view linking the temporary annual changes in illiteracy (Short-Run) and their continuous effects on poverty (Long-Run), while illustrating the speed at which deviations can be corrected, making the results usable in developing sustainable educational and social policies to reduce poverty.

Although the simple model shows a negative correlation between illiteracy and poverty, the ARDL model indicates a positive long-term association, suggesting that higher illiteracy rates are linked with higher poverty levels under the model’s dynamic structure. This reflects the fact that increased illiteracy leads to a sustained rise in poverty rates over the long term. Furthermore, the time delay analysis shows that the delayed effect of illiteracy represents the accumulation of educational deficits in society, increasing poverty before reaching long-term equilibrium, which reflects the structural and social factors of the Egyptian economy.

## Discussion and conclusion

### Discussion

The empirical findings of this study indicate that the relationship between illiteracy and poverty is dynamic rather than static. The simple linear regression model initially suggests a negative relationship between the two variables; however, this result must be interpreted with caution due to the presence of strong autocorrelation in the error terms and violations of the classical regression assumptions. Consequently, the static model fails to capture the delayed and cumulative effects of illiteracy on poverty.

In contrast, the ARDL model provides a more reliable and economically meaningful representation of this relationship. The dynamic estimates reveal that increases in illiteracy rates are associated with higher levels of poverty in both the short and long run. The positive long-run coefficient indicates that illiteracy contributes to structural poverty by constraining human capital formation, limiting labor market participation, and reducing long-term income opportunities.

The short-run dynamics further demonstrate that the impact of illiteracy on poverty is cumulative and unfolds gradually over time. The effects of educational deprivation are not immediately visible but tend to appear progressively at the societal and economic levels. This confirms that deficiencies in education can create persistent poverty traps.

These findings are consistent with previous studies that highlight the strong linkage between education and poverty reduction. Several empirical studies have demonstrated that higher literacy levels improve employment prospects, increase productivity, and enhance income opportunities (Pal, 2024; Galor & Zeira, 1993). Therefore, improving educational outcomes represents a fundamental mechanism for reducing poverty and fostering sustainable economic development.

However, the strength of this relationship may vary depending on regional economic conditions and socioeconomic structures. Future research could therefore explore this relationship across different regions and time periods in order to provide more comprehensive evidence.

Overall, the results emphasize the importance of implementing sustainable educational policies aimed at reducing illiteracy rates. Enhancing literacy levels can gradually reduce poverty while simultaneously promoting long-term economic and social stability.

### Conclusion and policy implications

This study examined the relationship between illiteracy and poverty in Egypt using both a simple linear regression model and a dynamic ARDL framework over the study period.

**First**, the results indicate that the ARDL model provides a more appropriate framework for capturing the dynamic relationship between illiteracy and poverty compared with the simple linear regression model. While the simple regression offers preliminary insights, it fails to account for short-run adjustments and long-run dynamics, as reflected in diagnostic limitations such as autocorrelation.

**Second**, the empirical findings reveal a strong association between illiteracy and poverty in Egypt during the study period. The ARDL(1,2) model explains a substantial proportion of the variation in poverty rates, with an explanatory power of approximately 92.9%. Although this relationship does not necessarily imply direct causality, the results highlight the important role of illiteracy in explaining fluctuations in poverty levels.

**Third**, the long-run estimates indicate a positive association between illiteracy and poverty, suggesting that higher illiteracy rates are linked to higher poverty levels within the dynamic structure of the model. In addition, the bounds testing procedure confirms the presence of both short-run and long-run equilibrium relationships between the variables, supporting the suitability of the ARDL framework for analyzing this relationship.

**Finally**, diagnostic tests further confirm that the estimated ARDL model is econometrically reliable and does not suffer from major specification problems such as autocorrelation, heteroskedasticity, or non-normality of the residuals.

From a policy perspective, the findings suggest that reducing illiteracy is closely associated with both short-term improvements and more persistent long-term reductions in poverty. This highlights the importance of prioritizing inclusive and sustainable education policies as a central component of poverty alleviation strategies in Egypt. Strengthening investment in educational infrastructure, improving teacher training, ensuring the effective implementation of compulsory education, and aligning educational outcomes with labor market requirements are essential policy priorities.

An integrated policy approach that combines institutional planning, adequate financial resources, and quality-oriented educational reforms can enhance human capital formation and contribute to long-term economic and social stability.

### Limitations of the study

Despite the contributions of this study, some limitations should be acknowledged. The analysis relies on a relatively small sample size and focuses on a single explanatory variable. Future research may therefore incorporate additional socioeconomic determinants and apply more advanced econometric techniques, such as nonlinear ARDL or threshold regression models, to examine potential nonlinear or asymmetric relationships between education and poverty.

Future research may extend the present analysis by incorporating additional economic and social variables and applying more advanced econometric techniques. Expanding the scope of the study to panel data or cross-country contexts could further enhance the robustness and generalizability of the findings and provide deeper insights into the education–poverty nexus in developing economies. Such extensions may also allow for international comparisons and a broader understanding of the dynamic interactions between education and poverty across different socioeconomic environments.

## Data Availability

The code used in this study is available at Zenodo: https://doi.org/10.5281/zenodo.19248884The data used in this study are publicly available and were obtained from official and reliable sources. Specifically, the data on illiteracy rates were collected from the Central Agency for Public Mobilization and Statistics (CAPMAS) and the World Bank Database, while the data on poverty rates were retrieved from the World Bank and the United Nations Human Development Reports. All data are freely accessible through the official websites of the mentioned organizations. The empirical analysis was conducted using EViews 12 (Version 12,https://www.eviews.com/v12/v12.html), which was employed to perform the econometric estimations and statistical analysis.
